# Analysis of neuroendoscopy for the treatment of macroadenomas and giant pituitary adenomas

**DOI:** 10.3389/fsurg.2022.956345

**Published:** 2022-08-11

**Authors:** Junyong Wu, Binbin Zhang, Dongqi Shao, Shuxin Ji, Yu Li, Shan Xie, Zhiquan Jiang

**Affiliations:** ^1^ Department of Neurosurgery, The First Affiliated Hospital of Bengbu Medical College, Bengbu, China; ^2^ Shandong University of Traditional Chinese Medicine, Jinan, China

**Keywords:** neuroendoscopy, transnasal butterfly approach, giant pituitary adenoma, pituitary surgery, macroadenoma

## Abstract

**Objective:**

This study investigated the use and effectiveness of endoscopic transnasal, transsphenoidal surgery, a minimally invasive method for the treatment of macroadenomas and giant pituitary a denomas, in a medical setting. The surgical results of 429 patients who received neuroendoscopic treatment of macroadenomas or giant pituitary adenomas were evaluated, and the experiences and lessons learned from treatment complications were assessed.

**Patients and methods:**

From January 2012 to December 2021, 429 patients with macroadenomas or giant pituitary adenomas, including 60 patients with giant adenomas (diameter ≥4 cm) and 369 patients with macroadenomas (diameter 1–4 cm), received a 3D head CT, a MRI with contrast enhancement, and an endocrinology examination prior to surgery. Preoperative clinical and radiological features, visual measurements, hormone levels, length of stay, length of surgery, postoperative stay, visual and hormone outcomes, resection range, complication and recurrence rates, and routine patient information were recorded. The patients were followed up for 6–72 months (median = 40 months).

**Results:**

Of 429 patients with macroadenomas or giant pituitary adenomas who received neuroendoscopic treatment, 348 (81.12%) had gross-total resections (GTR), 53 (12.35%) had near-total resections (NTR), and 28 (6.53%) had subtotal resections. There were 138 cases of post-operative diabetes insipidus (32.17%), including 7 cases of permanent diabetes insipidus (1.63%), 16 cases of nasal hemorrhage (3.73%), 39 cases of intraoperative cerebrospinal fluid leakage (9.09%), 4 cases of intracranial infection (0.9%), 16 cases of hypophysis (3.7%), and 15 cases of anosmia (3.50%). The clinical symptoms and endocrinology indices of the patients improved after surgery, and all patients were discharged 5–18 days (8.36 ± 2.65) postop.

**Conclusion:**

Neuroendoscopy is a safe operation with a short recovery period and hospital stay and is thus an effective method to treat macroadenomas and giant pituitary adenomas. Preoperative evaluation and prediction can help to accurately address possible intraoperative situations and improve GTR.

## Introduction

Pituitary adenomas are benign tumors of the anterior pituitary that lack classical oncogenic mutations. Disrupted cell cycle control and growth factor signaling may play a role in their pathogenesis and natural history (1). These adenomas represent approximately 15% of all intracranial adenomas, having the third highest incidence rate (2). A recent study also indicates that the prevalence rate of pituitary adenomas has increased from 7.5–15 to 77.6 per 100,000 persons (2). Pituitary adenomas can cause serious health issues among patients.

Medical, surgical, and/or radiosurgical treatments are used for pituitary adenomas, depending on the clinical status and size of the adenoma at the time of presentation (3). In pituitary adenoma patients experiencing clinical symptoms, surgical resection remains the most used clinical treatment. However, because of the irregular shape of some adenomas and important neurovascular involvement, the total resection rate of pituitary adenomas is low and recurrence is common (4). Surgery can be particularly challenging if the pituitary adenoma is a macroadenoma or giant pituitary adenoma. The traditional treatment for these adenomas is craniotomy and, if the adenoma has broken through the diaphragmatic sellae and the microscopic field of view is limited, brain tissue retraction is required (5). Postoperatively, patients are at risk for severe reactions, complications, and long hospital stays.

Neuroendoscopy, a technology that has developed rapidly over the past two decades, has been increasingly used for the treatment of pituitary adenomas (6, 7). While the efficacy of endoscopic intranasal sphenoidal surgery for macroadenomas and giant pituitary adenomas has been widely reported, the surgical cases and time spans explored by these studies have some important limitations (8–10).

The current study analyzes retrospective data from 429 pituitary adenoma patients who were treated at the Department of Neurosurgery of the First Affiliated Hospital of Bengbu Medical College, China from January 2012 to December 2021. A total of 429 patients met the criteria for butterfly macroadenoma and giant pituitary adenoma surgery, using a neuroendoscopic transnasal approach. The relationships between the surgical resection rate, Knosp classification, adenoma size, operation time, and adenoma-related complications were assessed. The advantages of transsphenoidal endoscopic resection of macroadenomas and giant pituitary adenomas were analyzed and discussed.

## Materials and methods

### Clinical materials

This study was approved by the Ethics Committee of the First Affiliated Hospital of Bengbu Medical College. The patients were treated in the Neurosurgery Department of the First Affiliated Hospital of Bengbu Medical College, China, from January 2012 to December 2021. 3D CT, MRI with contrast enhancement, and endocrine examinations were performed before surgery, and preoperative clinical and radiological characteristics, visual and hormonal outcomes, resection range, operation duration, postoperative discharge time, complications, recurrence rate and patient routine information were recorded and analyzed. Histopathological and immunohistochemical analyses were used to confirm the diagnosis of pituitary adenomas and assess multiple pituitary hormone levels. During data compilation, 429 patients met the radiological definitions of macroadenomas (1 ≤ D < 4 cm) and giant adenomas (≥4 cm) in addition to the criteria required for the neuroendoscopic transnasal surgical approach for butterfly macroadenoma and giant pituitary adenoma surgeries (11). Medical and nursing conditions remained consistent for all patients. Those patients with an adenoma diameter <1 cm or with incomplete follow-up records were excluded from the final analysis. All included cases were followed up for at least 6 months. Patient and adenoma characteristics are summarized in Table 1.

**Table 1:** Patient demographics and adenoma characteristics (*N *= 429).

Demographics	*N*	%
Male	195	45.45%
Female	234	54.55%
Mean age (years)	50.72 (8–78)	
LOS (length of stay)	16.9 (8–32)	
Postoperative hospital stay	8.4 (4–24)	
Diameter (average ± SD) (mm)	(26.57 ± 10.28)	
10–19	128	29.84%
20–29	131	30.54%
30–39	110	25.64%
>40	60	13.98%
Knosp classification		
Grade 0	129	30.1%
Grade 1	116	27.1%
Grade 2	96	22.4%
Grade 3A	51	11.8%
Grade 3B	27	6.3%
Grade 4	10	2.3%
Preoperative clinical signs and symptoms		
Visual field defects	278	64.80%
Anterior pituitary insufficiency	85	19.81%
Headache	159	37.06%
Drowsiness	5	1.17%
Treatment		
Endoscopic transnasal transsphenoidal surgery	429	100%
Surgical complications		
CSF leak	39	9.09%
Intracranial infection	4	0.93%
Loss of smell	15	3.50%
Diabetes insipidus	138	32.17%
Hypopituitarism	16	3.70%
Epistaxis	16	3.70%
Proliferation		
Nonproliferative	359	83.68%
Proliferative	70	16.32%

### Surgical methods

A neurosurgeon with >15 years of experience performed neuroendoscopy on all patients. The surgical objectives were to (1) achieve maximal resection and remission of symptoms with the least disturbance to neural and vascular structures and (2) maintain or reinstate endocrine function.

**Figure 1:**
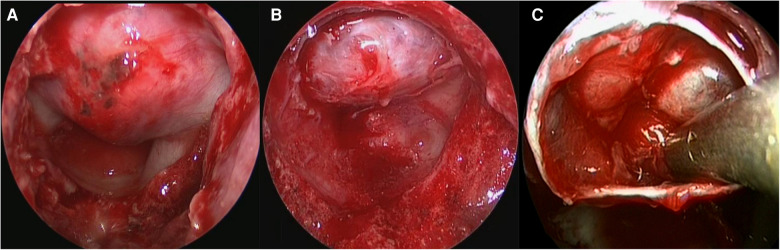
(**A–C**) Neuroendoscopy for the treatment of macroadenomas and giant pituitary adenomas. the full floor could be observed in the sphenoid sinus (**A**), The diameter of the sellar bottom bone window was ground to 1–2 cm (**B**), the endoscope was extended into the adenoma cavity to explore and remove the residual adenoma (**C**).

A transsphenoidal neuroendoscopic procedure was used to remove pituitary adenomas from a single (usually the right) nostril. During the procedure patients were supine with the head tilted posteriorly at 15°. Following induction of general anesthesia, the nasal mucosa and skin of the surgical site were disinfected, and a middle turbinate and septum approach was taken with the endoscope angled at 30°. After covering the nasal mucosa with an epinephrine-soaked cotton pad, the nasal turbinates were lateralized to expand the surgical space. The right pedicled nasoseptal flap was partially resected, stored inferior to the surgical channel, and fully harvested if an intraoperative cerebrospinal (CSF) leak occurred. A high-speed drill or osteotome was used to open the sphenoid sinus, and the sellar floor was removed so that the full floor could be observed in the sphenoid sinus (Figure 1A). The diameter of the sellar bottom bone window was ground to 1–2 cm (Figure 1B), the intrasellar aneurysm was treated by puncture, and the adenoma was removed using a pituitary curette and attractor. The field was intermittently flushed with saline, and any residual adenoma was observed in real-time during endoscopic resection. Residual lesions in the cavernous sinus were removed under direct observation. Intrasellar and suprasellar adenomas were completely resected, and the endoscope was extended into the adenoma cavity to explore and remove any residual adenoma (Figure 1C). After hemostasis, the skull base was reconstructed using autologous tissue and artificial materials.

### Data analysis

Continuous variables are presented as the mean, range, and median, and categorical data are presented as total counts and proportions. Demographic, clinical, radiological, and intraoperative adenoma characteristics of the resection range were analyzed using the Chi-square test. All statistical analyses were performed using SPSS version 22.0 (IBM Corporation), and a *p*-value of <0.05 was considered statistically significant.

## Results

### Patient characteristics

The male-to-female ratio of the 429 patients included in this study was 0.83:1. The median age was 53 years (range 8–78 years), and most cases were nonfunctional pituitary adenomas (NFPA) (*n* = 277). The majority of functional pituitary adenoma (FPA; *n* = 152) cases were those induced by the overproduction of growth hormone (*n* = 34; 7.92%) and prolactin (*n* = 110; 25.64%), followed by those induced by corticotropin (*n* = 6; 1.40%) or thyrotropin (*n* = 2; 0.47%). The Ki-67 labeling index was ≥5% in 31 patients (7.13%), <3% in 325 patients (75.76%), and 3%–5% in 74 patients (17.11%). P53 staining was positive in 51 patients (11.89%), negative in 359 patients (83.68%), and weak in 19 patients (4.43%) (Table 2). The most common symptoms before surgery included impaired visual acuity and visual field defects (*n* = 278; 64.8%), headache (*n* = 159; 37.06%), and endocrine-related indications (*n* = 85; 19.81%).

**Table 2:** Pathological characteristics.

Cell types	*N* (%)
Non-functioning	277 (64.57)
Prl	110 (25.64)
GH	34 (7.92)
ACTH	6 (1.40)
TSH	2 (0.47)
Ki-67	
<3%	325 (75.76)
3%–5%	74 (17.11)
>5%	31 (7.13)
P53	
Negative	359 (83.68)
Positive	51 (11.89)
Weak	19 (4.43)

### Imaging classification

The revised Knosp classification for “invasion of cavernous sinus space in pituitary adenoma”, devised by Micko et al. (12), was used for all cases. Most patients (*n* = 129; 30.1%) received a Knosp Grade of 0. Adenomas in 116 patients (27.1%) were classified as Grade 1, 92 (21.4%) classified as Grade 2, 35 (8.16%) classified as Grade 3A, 17 (3.9%) classified as Grade 3B, and 10 (2.3%) classified as Grade 4. All cases were either macroadenomas (diameter >1 cm) or giant pituitary adenomas (>4 cm), with a mean diameter of 2.66 ± 0.51 cm.

### Results of excision range

**Figure 2:**
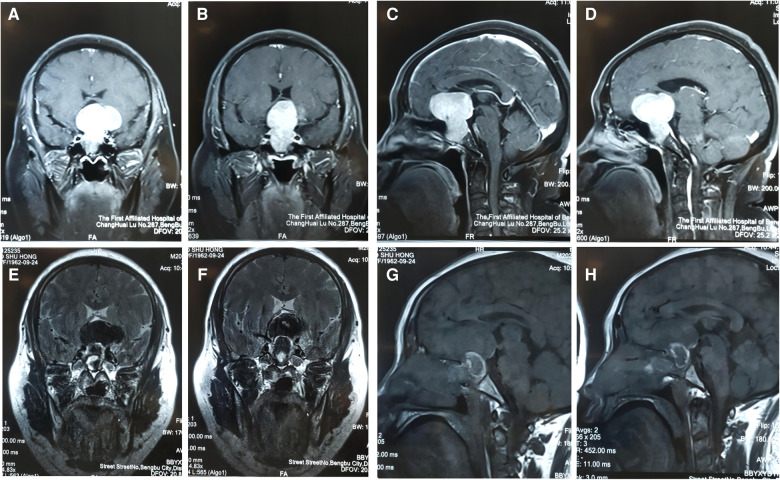
(**A–H**): This was a 50-year-old female patient who was admitted to hospital after the discovery of a pituitary tumor due to clinical manifestation. Preoperative MRI of a special giant pituitary adenoma (**A–D**). Postoperative MRI (**E–H**).

A total resection was performed for 348 cases (81.12%), had available preoperative and postoperative MRI studies (Figure 2) a near total resection was performed for 53 cases (12.35%), and a major resection was performed for 28 cases (6.53%). All of them had available preoperative and postoperative MRI studies (Figure 2). There were 138 cases of postoperative diabetes insipidus (DI) (8.86%), including 7 cases of permanent DI (1.63%), 16 cases of nasal hemorrhage (3.73%), 39 cases of intraoperative CSF leakage (9.09%), 4 cases of intracranial infection (0.9%), 16 cases of hypophysis (3.7%), and 15 cases of anosmia (3.50%). Patient clinical symptoms and endocrinological indicators improved during the follow-up period (Table 3).

**Table 3:** Preoperative symptoms that improved after surgery.

Symptoms	Improved/Total (%)
Preoperative symptoms	
Visual field defects	277/278 (99.6)
Anterior pituitary insufficiency	44/85 (51.8)
Headache	131/159 (82.4)
Drowsiness	5/5 (100)
Syndrome	
CSF leak	39/39 (100)
Intracranial infection	4/4 (100)
Loss of smell	10/15 (66.7)
Diabetes insipidus	131/138 (94.9)
Hypopituitarism	9/16 (56.3)
Epistaxis	16/16 (100)

### Factors for the extent of resection

The factors influencing adenoma resection are summarized in Table 4. The nature and shape of the adenoma significantly affected the resection range (*p* < 0.01). For example, GTR was easier to obtain for giant pituitary adenomas with a Knosp Grade of 0–1.

**Table 4:** Factors influencing adenoma resection.

Tumor type	GTR (%)	NGTR* (%)	*p*-value
Knosp classification			
Grade 0	114 (88.37)	15 (11.63)	<0.01
Grade 1	103 (88.79)	13 (11.21)	
Grade 2	75 (79.79)	19 (20.21)	
Grade 3A	35 (68.64)	18 (31.37)	
Grade 3B	17 (62.96)	10 (37.04)	
Grade 4	4 (40)	6 (60)	
Diameter (mm)			
10–19	114 (89.06)	14 (10.94)	<0.01
20–29	117 (89.31)	14 (10.69)	
30–39	80 (76.19)	25 (23.81)	
>40	37 (56.92)	28 (43.08)	
Hormone secretion			
NFPA	221 (82.85)	46 (17.25)	0.261
FPA	127 (78.43)	35 (21.67)	
Operative history			
Primary	339 (87.59)	48 (12.41)	<0.01
Recurrent	9 (21.92)	33 (78.18)	
Tumor shape			
Rounded	289 (92.04)	25 (7.96)	<0.01
Dumbbell	35 (53.03)	31 (46.97)	
Multilobular	24 (48.98)	25 (51.02)	

* Not total resection, including near total resection and a major resection.

## Discussion

A pituitary adenoma is a prevalent brain tumor that often invades the peripheral nerves, blood vessels, and cavernous sinus or suprasellar or paracellar regions. GTR of pituitary adenomas is technically challenging, even for highly experienced neurosurgeons (13). Macroadenomas are defined as adenomas that are >1 cm in diameter, and giant pituitary adenomas are defined as those with a diameter >4 cm (3). Giant pituitary adenomas are estimated to account for 5%–10% of all pituitary adenomas (14). Surgical resection, which is used to restore normal pituitary function, decompresses nerves and blood vessels with minimal damage to surrounding tissues and is considered the first-line treatment for pituitary adenoma. Since 1990, transsphenoidal neuroendoscopic surgery has been widely used because of its ability to enlarge and improve visual clarity of the surgical field.

Both transcranial and transsphenoidal approaches can be used to remove macroadenomas and giant pituitary adenomas, but the transsphenoidal approach is the preferred choice for resection (15). In recent years, the use of endoscopic surgery for pituitary adenomas has significantly increased in the United States (16), and the use of microsurgery has decreased. Møller et al. (17) found that patients undergoing endoscopic surgery for pituitary adenomas had better surgical outcomes and fewer complications than those undergoing a microsurgical approach. This is partially due to the better light sources and high-definition cameras used in endoscopic technology, which have improved visualization and provided a panoramic view of the sellar, paracellar and suprasellar areas. Many studies have demonstrated the possible superiority of endoscopy over traditional microscopy for both functional and nonfunctional pituitary adenomas.

For giant pituitary adenomas, especially those that are nonfunctional, total resection remains difficult. This may be because nonfunctional pituitary adenomas are difficult to detect until they become large enough to compress surrounding tissues (18). However, there has been success with endoscopic transsphenoidal and endoscopic combined resection of giant pituitary adenomas protruding into the third ventricle, as well as with endoscopy combined with a transsphenoidal suprasellar keyhole approach for the treatment of complex paraselellar pituitary adenomas (19, 20). Thus, some researchers believe that endoscopic transsphenoidal resection of giant pituitary adenomas is superior to microsurgery. During the resection of a giant adenoma, the order of surgical procedures is critical to prevent the premature decline of the sella turcica diaphragm, which can affect the adenoma resection, puncture the sella turcica diaphragm and cause CSF leakage. In general, it is advised to begin by removing the lower adenoma and cut both sides before removing the upper adenoma. The adenoma should be removed gently with a pituitary curette to prevent the rupture of the sellar diaphragm and potential CSF leakage. In addition, the adenoma should be removed *in situ* using an adenoma suction apparatus or a curette. The use of forceps should be avoided, as this can lead to intracranial hemorrhage or vision changes. Patients with partial resection of the pituitary adenoma are at risk of postoperative adenoma residual bleeding; thus, intraoperative hemostasis should be accurate, and patients should be watched closely after surgery. If a patient shows symptoms of vision loss or increased intracranial pressure, the head CT should be reviewed in a timely manner to ensure an accurate diagnosis, and an emergency craniotomy should be performed if necessary.

As a result of its optical lighting characteristics, the endoscopy angle, and the fisheye effect, neuroendoscopy conveniently reveals lesions at a closer range and higher exposure than the traditional transsphenoidal approach. While poor sphenoidal sinus gasification and giant pituitary adenomas, including the dumbbell type, fiber type, protrusion into the third ventricle, and invasion into the cavernous sinus, were previously considered contraindications of transsphenoidal surgery, this is no longer the case. A safe endoscopic resection can be accomplished for cases of sphenoid sinus gasification and poor pituitary adenoma because the retractor does not have to be used for full exposure and the eye shot is open, allowing enough space in the nose to use a high-speed grinding drill. Bone grinding can be conducted in all directions, and the depth and direction can be adjusted to identify the sphenoid sinus by various anatomical landmarks to ensure that the surgery proceeds in the correct direction. For macroadenomas and multilobular, fibrous, and dumbbell giant pituitary adenomas that are difficult to resect, invasion of the instrument into the subarachnoid space may cause CSF leakage and damage to the optic nervous system and adjacent blood vessels that complicate the operation. Existing surgical options include combined or staged transsphenoidal-transcranial approaches and staged transsphenoidal resections after the residual adenoma descends into the sella (21). Lumbar infusions or air injection to encourage descent of the suprasellar adenoma component have also been used (22). For the special pituitary adenoma described earlier, bone and dura resection at the sella plane were used in addition to opening the sella to provide a double surgical corridor. First, an endosellar, extraarachnoidal corridor was created to debulk the sellar component of the adenoma. Second, a suprasellar transarachnoidal corridor was created to debulk the suprasellar component of the lesion and sharp dissect the adenoma capsule from the overlying parasellar cisterns and optic apparatus under direct visualization. This was performed to avoid adenoma residue and brain tissue damage.

Thus, for macroadenomas and giant pituitary adenomas with a special shape, endoscopic surgery has obvious advantages over microsurgery or craniotomy. This technique may provide a greater resection area and prevent blind curettage of the suprasellar components, significantly reducing the risk of neurovascular injury. Prolonging the intranasal approach can also help to expose large lesions behind the dural opening, helping surgeons to avoid retraction of neurovascular structures. This ensures that all dissection is performed on the surface of the adenoma, without risking brain damage or traction of the olfactory tract.

Recent reports of surgical complications are consistent with those described in the current study (23–29). CSF leakage, for example, is a common complication of transsphenoidal neuroendoscopic resection (30), and there were 39 such cases in this series (9.1%). This complication is usually the result of surgical injury and adenoma invasion, especially for giant pituitary adenomas with anterior cranial fossa dilation (31). In the current study, most CSF leakage occurred during adenoma resection. This can be prevented by removing macroadenomas and giant adenomas along the edge of the sellar septum to prevent them from collapsing prematurely and damaging the arachnoid of the suprasellar cistern. If the diaphragmatic sella is ruptured intraoperatively, three layers of sellar bottom repair is often required. First, the adenoma cavity is filled with fat taken from the patient's outer thighs or lower abdomen. Then, artificial dura matter is used to cover the bottom of the sella turcica. This artificial dura matter is covered with autologous muscle and surrounded with medical adhesive to bind it. Finally, the artificial dura and muscle layer is covered, and the periphery is glued with medical adhesive to prevent additional leakage after the repair. If the sella turcica diaphragm is not damaged during the operation, it is not usually necessary to repair the bottom with fat and muscle. Rather, a gelatin sponge, quick gauze, and artificial dura mater can be used. Prior studies have reported a significant correlation between CSF leakage and postoperative intracranial infection, a common cause of death for patients with neuroendoscopic pituitary adenomas (32). Perioperative use of antibiotics and avoiding an excessively long operation time are important measures to reduce intracranial infection. Indeed, the probability of intracranial infection doubles when the operation time exceeds 3–4 h (33).

DI was the most common surgical complication of endoscopic resection of macroadenomas and giant pituitary adenomas in this study; however, only 7 cases eventually developed permanent DI (5.07%). The type and location of pituitary adenomas are related to the occurrence of DI after surgery (34). For postoperative DI, treatment is focused on reducing urine output, replacing fluid loss, maintaining normal plasma osmotic pressure, and reducing or stopping the use of osmotic diuretics. For patients with mild DI with a urine volume of 3,000–5,000 ml in 24 h, oral camassia equine should be given to observe the curative effect. For those with moderate DI with a urine volume of 5,000–6,000 ml within 24 h, intramuscular injection of 6U pituitrin should be initiated and repeated after 12 h, with the amount being adjusted based on changes in urine volume. For those with severe DI with a 24 h urine volume >6,000 ml, 6U pituitrin should be tried first, and repeated at an interval of 8–12 h if the effect is not obvious. The times of pituitrin administration can be increased or the vasopressin tannate can be changed. The initial dose is 0.2 ml, and blood electrolytes can be monitored to prevent electrolyte disorder.

MRI is the most important method for follow-up after surgical treatment of pituitary adenomas. The primary use of postoperative MRI is to evaluate the effectiveness of surgery. However, even after the removal of the pituitary adenoma, the mass may not initially appear smaller on an MRI as a result of fillings, postoperative debris, mucosal thickening, and blood accumulation. These postoperative features begin to disappear and the adenoma volume gradually decreases over several months (35). Thus, it is generally recommended that patients have a second MRI within 6 months after surgery, and approximately every 6 months thereafter. Follow-up after surgical treatment of pituitary macroadenoma should also include postoperative ophthalmologic evaluation after 1–2 weeks, and follow-up evaluation at 1 and 2 years to evaluate the final effect of surgical treatment on visual functioning (36). An ophthalmologist at our hospital usually provides visual acuity and visual field examinations before and after surgery and at 3, 6, 12, and 24 months of follow-up. If the patient's visual acuity improves and stays the same during follow-up, the prognosis is good. For patients with extrasellar residues, actively administering radiotherapy to reduce the incidence of recurrence or closely observing patients and only administering radiotherapy to those who do develop recurrence remains controversial (37). The potential side effects of radiation therapy and the development of hypopituitarism need to be balanced against the risk of adenoma growth and vision loss. We believe that, under the guarantee of strict postoperative follow-up, the appropriate patients can be treated conservatively if there are no obvious postoperative symptoms of compression. If clinical symptoms develop, radiation therapy or reoperation may be required.

GTR is the optimal surgical outcome for macroadenoma and giant pituitary adenomas. We identified independent risk factors for resection scope to aid in the development of an appropriate surgical strategy. The size of the adenoma and the invasiveness of the giant pituitary adenoma into surrounding structures are key factors limiting the scope of resection. The current study found that an increase in adenoma diameter and Knosp grade decreased GTR opportunities. Therefore, both maximum diameter and Knosp grading were independent factors for the extent of resection. Sanmillan et al. (38) found that in 294 patients with pituitary adenoma, adenoma volume and Knosp grade were considered independent risk factors for resection scope, with Knosp grade having a greater impact. Similarly, we found that some giant pituitary adenomas with a low Knosp grade could be satisfactorily removed despite their large size. Thus, determining whether the pituitary adenoma invades the cavernous sinus is critical for surgical planning, and adenoma size can provide supplementary information. Additional improvements in surgical instruments, computer simulations, and endoscopes may further improve surgical resection rates and reduce the incidence of complications.

### Limitations

The limitations of this study are primarily related to its retrospective design, lack of randomization, and the surgeon's assessment of the results. The results only present the experience provided from a single center with specific surgical techniques and protocols. In addition, this study only focused on patients undergoing endoscopic transnasal transsphenoidal surgery, which may lead to some epidemiological biases.

## Conclusion

This study showed that the resection rate of pituitary adenomas under endoscopy was proportional to the size and Knosp grade of the pituitary adenoma. Preoperative evaluation of the Knosp grade helps identify situations where endoscopic approaches may be inadequate, allowing for more accurate treatment and preparation for possible serious complications. The findings shown here illustrate that neuroendoscopic transsphenoidal resection of pituitary adenomas is a safe and effective surgical method for pituitary adenomas with a clear surgical field, wide exposure range, and high adenoma resection rate and result in a lower rate of postoperative complications, quick postoperative recovery, and a short hospital stay.

## Data Availability

The raw data supporting the conclusions of this article will be made available by the authors, without undue reservation.
